# (3*E*)-3-[4-(Dimethyl­amino)­phen­yl]-1-(4-hy­droxy­phen­yl)prop-2-en-1-one

**DOI:** 10.1107/S1600536810024050

**Published:** 2010-06-26

**Authors:** Aurangzeb Hasan, Nadeem Akhtar, Nordin Hj Lajis, Aqilah Fasihah Binti Rusli, Kong Mun Lo

**Affiliations:** a Department of Chemistry, University of Malaya, 50603 Kuala Lumpur, Malaysia; b Laboratory of Natural Products, Institute of Bioscience, University Putra Malaysia, 43400 UPM Serdang, Selangor Darul Ehsan, Malaysia; c Department of Chemistry, Faculty of Science, University Putra Malaysia, 43400 UPM Serdang, Selangor Darul Ehsan, Malaysia

## Abstract

The asymmetric unit of the title compound, C_17_H_17_NO_2_, contains two crystallographically independent mol­ecules. Both mol­ecules adopt a *trans* configuration about the C=C bond, with the C—C=C—C fragments in the two mol­ecules twisted in opposite directions [torsion angles of 174.2 (2) and −175.8 (2)°]. The two benzene rings in each of the mol­ecules make dihedral angles of 20.21 (6) and 48.64 (4)°. In the crystal, adjacent mol­ecules are linked by O—H⋯O hydrogen bonds into infinite polymeric chains.

## Related literature

For the biological activity of chalcones, see: Sortino *et al.* (2007 ▶); Katsori & Hadjipavlou-Litina (2009 ▶). For the use of chalcones as precursors in the preparation flavonoids, see: Avila *et al.* (2008 ▶). For the crystal structures of related chalcone derivatives, see: Liu *et al.* (2002 ▶); Fronczek *et al.* (1987 ▶).
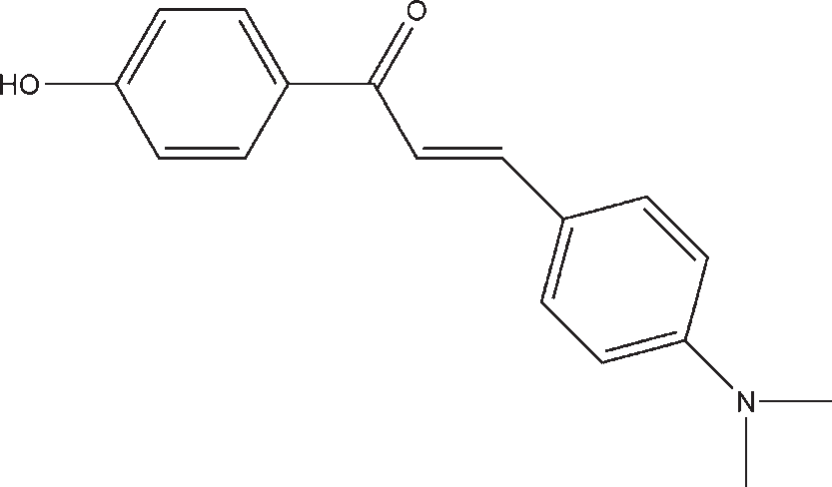


## Experimental

### 

#### Crystal data

C_17_H_17_NO_2_
*M* *_r_* = 267.32
Monoclinic, 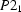

*a* = 6.3070 (1) Å
*b* = 29.5285 (6) Å
*c* = 7.3880 (2) Å
β = 95.056 (1)°
*V* = 1370.56 (5) Å^3^
*Z* = 4
Mo *K*α radiation
μ = 0.09 mm^−1^
*T* = 100 K
0.48 × 0.24 × 0.16 mm

#### Data collection

Bruker APEXII CCD area-detector diffractometer
Absorption correction: multi-scan (*SADABS*; Sheldrick, 2008*a* ▶) *T*_min_ = 0.960, *T*_max_ = 0.987
8837 measured reflections
2756 independent reflections
2646 reflections with *I* > 2σ(*I*)
*R*_int_ = 0.020

#### Refinement

*R*[*F* ^2^ > 2σ(*F* ^2^)] = 0.032
*wR*(*F* ^2^) = 0.078
*S* = 1.13
2756 reflections
367 parameters
1 restraint
H-atom parameters constrained
Δρ_max_ = 0.17 e Å^−3^
Δρ_min_ = −0.26 e Å^−3^

### 

Data collection: *APEX2* (Bruker, 2008 ▶); cell refinement: *SAINT* (Bruker, 2008 ▶); data reduction: *SAINT*; program(s) used to solve structure: *SHELXS97* (Sheldrick, 2008*b* ▶); program(s) used to refine structure: *SHELXL97* (Sheldrick, 2008*b* ▶); molecular graphics: *X-SEED* (Barbour, 2001 ▶); software used to prepare material for publication: *publCIF* (Westrip, 2010 ▶).

## Supplementary Material

Crystal structure: contains datablocks I, global. DOI: 10.1107/S1600536810024050/fj2315sup1.cif

Structure factors: contains datablocks I. DOI: 10.1107/S1600536810024050/fj2315Isup2.hkl

Additional supplementary materials:  crystallographic information; 3D view; checkCIF report

## Figures and Tables

**Table 1:** Hydrogen-bond geometry (Å, °)

*D*—H⋯*A*	*D*—H	H⋯*A*	*D*⋯*A*	*D*—H⋯*A*
O4—H4*O*⋯O1^i^	0.82	1.85	2.670 (2)	173
O2—H2*O*⋯O3^ii^	0.82	1.85	2.659 (2)	169

Symmetry codes: (i) 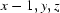; (ii) 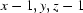.

**Fig. 1.**
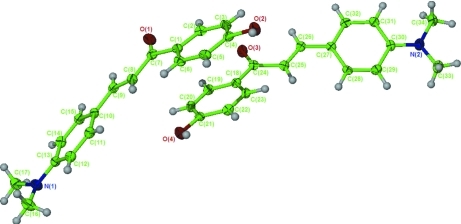
The molecular structure of 3E-(4-dimethylaminophenyl)-1-(4'-hydroxyphenyl)-prop-2-en-1-one showing 70% probability displacement ellipsoids and the atom numbering. Hydrogen atoms are drawn as spheres of arbitrary radius.

**Fig. 2.**
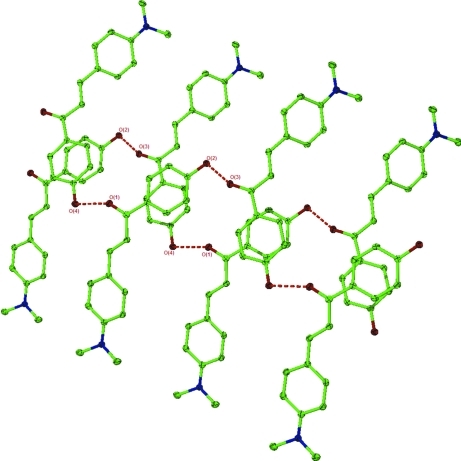
Crystal packing showing the hydrogen bonding interactions in the molecules.

## supplementary crystallographic information

### Comment

Chalcone is a unique template that is associated with several biological activities. The radical quenching properties of the phenolic groups present in many chalcones have raised interest in using the compounds or chalcone rich plant extracts as drugs or food preservatives [Sortino, *et al.* (2007); Katsori, *et al.* (2009)]. Chalcones constitute an important group of natural product and serve as precursors for the synthesis of different classes of flavonoids, which are common substances in plants [Avila, *et al.* (2008)]. We report here a substituted chalcone derivative which is prepared from the condensation reaction of *p*-hydroxylacetophenone with 4-(*N,N*-dimethylamino)benzaldehyde. The crystal structure of this compound (common chemical name: 4-hydroxy-4'-dimethylaminochalcone) consists of two independent molecules which form polymeric chains as a result of intermolecular hydrogen bonding between the hydroxyl groups and carbonyl oxygen atoms of adjacent molecules (Fig. 2). In contrast, the related compounds, 2-hydroxy-4'-dimethylaminochalcone [Liu, *et al.* (2002)] and 2,4-dihydroxychalcone [Fronczek, *et al.* (1987)] are discrete molecules. In the title compound, the two asymmetric molecules adopt the *trans* configuration about the olefinic double bond with torsional angles of 174.2 (2)° and -175.8 (2)^o ^. In addition, the two benzene rings in both molecules are not co-planar, but makes a dihedral angle of 20.21 (6)° and 48.64 (4)°, respectively.

### Experimental

To a stirred solution of KOH (2.0 g, 45.6 mmol) in distilled water (2 ml) cooled in an ice bath, was added 10 ml of methanoic solution containing *p*-hydroxyacetophenone (g, 1 mmol) and 4-(*N,N*-dimethylamino)benzaldehyde (g, 1 mmol). The reaction mixture was stirred at room temperature for 24 h. The mixture was poured into ice-water (10 ml), adjusted to pH 5 - 6 with 1*M* HCl, and then extracted with ethyl acetate. The organic layer was successively washed with distilled water and saturated brine, dried over anhydrous sodium sulfate. The resulting filtrate was evaporated slowly at room temperature to obtain the yellow crystals.

### Refinement

Hydrogen atoms were placed at calculated positions (C–H 0.93 Å; O–H 0.82 Å) and were treated as riding on their parent atoms, with *U*(H) set to 1.2–1.5 times *U*~eq~(C). The absolute structure could not be determined from the X-ray analysis. 2266 Friedel pairs were therefore merged before the final refinement.

### Crystal data

**Table:**

C_17_H_17_NO_2_	*F*(000) = 568
*M**_r_* = 267.32	*D*_x_ = 1.295 Mg m^−^^3^
Monoclinic, *P*2_1_	Mo *K*α radiation, λ = 0.71073 Å
Hall symbol: P 2yb	Cell parameters from 4618 reflections
*a* = 6.3070 (1) Å	θ = 2.8–29.5°
*b* = 29.5285 (6) Å	µ = 0.09 mm^−^^1^
*c* = 7.3880 (2) Å	*T* = 100 K
β = 95.056 (1)°	Block, yellow
*V* = 1370.56 (5) Å^3^	0.48 × 0.24 × 0.16 mm
*Z* = 4	

### Data collection

**Table:**

Bruker APEXII CCD area-detector diffractometer	2756 independent reflections
Radiation source: fine-focus sealed tube	2646 reflections with *I* > 2σ(*I*)
graphite	*R*_int_ = 0.020
ω scans	θ_max_ = 26.0°, θ_min_ = 1.4°
Absorption correction: multi-scan (*SADABS*; Sheldrick, 2008a)	*h* = −7→7
*T*_min_ = 0.960, *T*_max_ = 0.987	*k* = −35→36
8837 measured reflections	*l* = −9→8

### Refinement

**Table:**

Refinement on *F*^2^	Primary atom site location: structure-invariant direct methods
Least-squares matrix: full	Secondary atom site location: difference Fourier map
*R*[*F*^2^ > 2σ(*F*^2^)] = 0.032	Hydrogen site location: inferred from neighbouring sites
*wR*(*F*^2^) = 0.078	H-atom parameters constrained
*S* = 1.13	*w* = 1/[σ^2^(*F*_o_^2^) + (0.0389*P*)^2^ + 0.282*P*] where *P* = (*F*_o_^2^ + 2*F*_c_^2^)/3
2756 reflections	(Δ/σ)_max_ = 0.044
367 parameters	Δρ_max_ = 0.17 e Å^−^^3^
1 restraint	Δρ_min_ = −0.26 e Å^−^^3^

### Special details

**Table:**

Geometry. All e.s.d.'s (except the e.s.d. in the dihedral angle between two l.s. planes) are estimated using the full covariance matrix. The cell e.s.d.'s are taken into account individually in the estimation of e.s.d.'s in distances, angles and torsion angles; correlations between e.s.d.'s in cell parameters are only used when they are defined by crystal symmetry. An approximate (isotropic) treatment of cell e.s.d.'s is used for estimating e.s.d.'s involving l.s. planes.
Refinement. Refinement of *F*^2^ against ALL reflections. The weighted *R*-factor *wR* and goodness of fit *S* are based on *F*^2^, conventional *R*-factors *R* are based on *F*, with *F* set to zero for negative *F*^2^. The threshold expression of *F*^2^ > σ(*F*^2^) is used only for calculating *R*-factors(gt) *etc*. and is not relevant to the choice of reflections for refinement. *R*-factors based on *F*^2^ are statistically about twice as large as those based on *F*, and *R*- factors based on ALL data will be even larger.

### Fractional atomic coordinates and isotropic or equivalent isotropic displacement parameters (Å^2^)

**Table:**

	*x*	*y*	*z*	*U*_iso_*/*U*_eq_	
O2	−0.0711 (2)	0.31769 (5)	0.0870 (2)	0.0206 (3)	
H2O	−0.1727	0.3108	0.0154	0.031*	
O1	0.6270 (2)	0.17288 (6)	0.3369 (2)	0.0228 (4)	
O4	−0.0471 (2)	0.14648 (5)	0.5680 (2)	0.0210 (3)	
H4O	−0.1537	0.1540	0.5035	0.031*	
O3	0.6359 (2)	0.29454 (6)	0.8222 (2)	0.0217 (4)	
C22	−0.0250 (3)	0.22737 (8)	0.6122 (3)	0.0178 (5)	
H22	−0.1639	0.2313	0.5614	0.021*	
C18	0.3052 (3)	0.25932 (7)	0.7460 (3)	0.0159 (4)	
C4	0.0430 (3)	0.27980 (8)	0.1369 (3)	0.0167 (4)	
C5	−0.0379 (3)	0.23644 (8)	0.1061 (3)	0.0176 (5)	
H5	−0.1749	0.2323	0.0510	0.021*	
C21	0.0628 (3)	0.18403 (7)	0.6272 (3)	0.0166 (4)	
C30	0.1868 (3)	0.50908 (7)	1.0211 (3)	0.0165 (4)	
C7	0.4345 (3)	0.16614 (8)	0.2975 (3)	0.0178 (5)	
C27	0.3502 (3)	0.42371 (8)	0.9189 (3)	0.0161 (4)	
C24	0.4395 (3)	0.29859 (8)	0.8066 (3)	0.0161 (4)	
C20	0.2715 (3)	0.17818 (8)	0.7049 (3)	0.0182 (5)	
H20	0.3292	0.1493	0.7188	0.022*	
C1	0.2959 (3)	0.20472 (8)	0.2383 (3)	0.0162 (4)	
C26	0.4387 (3)	0.38086 (8)	0.8657 (3)	0.0166 (4)	
H26	0.5806	0.3809	0.8407	0.020*	
C6	0.0875 (3)	0.19938 (8)	0.1583 (3)	0.0178 (5)	
H6	0.0324	0.1704	0.1399	0.021*	
C8	0.3399 (3)	0.12117 (8)	0.3178 (3)	0.0179 (5)	
H8	0.1998	0.1160	0.2723	0.021*	
C3	0.2483 (3)	0.28595 (7)	0.2200 (3)	0.0179 (5)	
H3	0.3007	0.3150	0.2428	0.021*	
C25	0.3352 (3)	0.34110 (7)	0.8492 (3)	0.0164 (4)	
H25	0.1907	0.3405	0.8656	0.020*	
C23	0.0940 (3)	0.26418 (8)	0.6726 (3)	0.0164 (4)	
H23	0.0332	0.2929	0.6646	0.020*	
C29	0.0770 (3)	0.46832 (8)	1.0495 (3)	0.0183 (4)	
H29	−0.0513	0.4693	1.1026	0.022*	
C10	0.3791 (3)	0.04249 (7)	0.4464 (3)	0.0165 (4)	
C32	0.4583 (3)	0.46418 (8)	0.8915 (3)	0.0183 (4)	
H32	0.5873	0.4631	0.8397	0.022*	
C19	0.3911 (3)	0.21555 (8)	0.7608 (3)	0.0175 (5)	
H19	0.5311	0.2116	0.8090	0.021*	
C28	0.1567 (3)	0.42723 (7)	1.0000 (3)	0.0164 (4)	
H28	0.0808	0.4010	1.0206	0.020*	
C2	0.3732 (3)	0.24881 (8)	0.2682 (3)	0.0178 (5)	
H2	0.5110	0.2531	0.3213	0.021*	
C9	0.4533 (3)	0.08742 (8)	0.4012 (3)	0.0176 (5)	
H9	0.5963	0.0935	0.4339	0.021*	
C13	0.2550 (3)	−0.04651 (7)	0.5389 (3)	0.0174 (5)	
C11	0.1743 (3)	0.02584 (8)	0.3928 (3)	0.0179 (4)	
H11	0.0777	0.0444	0.3259	0.021*	
C34	0.2379 (4)	0.59098 (8)	1.0528 (4)	0.0260 (5)	
H34A	0.3797	0.5867	1.1087	0.039*	
H34B	0.1724	0.6160	1.1095	0.039*	
H34C	0.2434	0.5972	0.9257	0.039*	
C12	0.1129 (3)	−0.01720 (7)	0.4367 (3)	0.0180 (5)	
H12	−0.0237	−0.0272	0.3987	0.022*	
C14	0.4592 (3)	−0.02979 (8)	0.5949 (3)	0.0189 (4)	
H14	0.5556	−0.0480	0.6641	0.023*	
C31	0.3797 (3)	0.50556 (8)	0.9388 (3)	0.0183 (4)	
H31	0.4552	0.5317	0.9162	0.022*	
C15	0.5175 (3)	0.01333 (7)	0.5481 (3)	0.0182 (5)	
H15	0.6541	0.0234	0.5857	0.022*	
C17	0.3609 (4)	−0.12290 (8)	0.6381 (3)	0.0230 (5)	
H17A	0.4533	−0.1263	0.5425	0.035*	
H17B	0.2955	−0.1515	0.6607	0.035*	
H17C	0.4421	−0.1128	0.7466	0.035*	
C16	−0.0076 (4)	−0.10756 (8)	0.5120 (4)	0.0268 (5)	
H16A	−0.1188	−0.0874	0.5420	0.040*	
H16B	−0.0294	−0.1368	0.5637	0.040*	
H16C	−0.0101	−0.1102	0.3823	0.040*	
C33	−0.0982 (4)	0.55599 (8)	1.1320 (4)	0.0258 (5)	
H33A	−0.2015	0.5508	1.0308	0.039*	
H33B	−0.1138	0.5862	1.1766	0.039*	
H33C	−0.1200	0.5347	1.2269	0.039*	
N2	0.1144 (3)	0.55029 (7)	1.0744 (3)	0.0235 (4)	
N1	0.1970 (3)	−0.08973 (6)	0.5844 (3)	0.0206 (4)	

### Atomic displacement parameters (Å^2^)

**Table:**

	*U*^11^	*U*^22^	*U*^33^	*U*^12^	*U*^13^	*U*^23^
O2	0.0188 (8)	0.0162 (8)	0.0260 (9)	0.0006 (6)	−0.0025 (6)	−0.0001 (7)
O1	0.0165 (7)	0.0225 (8)	0.0289 (9)	−0.0018 (6)	−0.0013 (6)	0.0043 (7)
O4	0.0201 (8)	0.0171 (8)	0.0248 (9)	−0.0012 (6)	−0.0036 (7)	0.0010 (7)
O3	0.0156 (7)	0.0207 (8)	0.0282 (9)	0.0017 (6)	−0.0018 (6)	−0.0032 (7)
C22	0.0146 (10)	0.0223 (12)	0.0160 (11)	0.0010 (9)	−0.0016 (8)	0.0000 (9)
C18	0.0157 (10)	0.0183 (11)	0.0136 (11)	0.0007 (9)	0.0011 (8)	0.0000 (9)
C4	0.0175 (10)	0.0184 (11)	0.0143 (10)	0.0009 (9)	0.0029 (8)	0.0013 (9)
C5	0.0153 (10)	0.0192 (11)	0.0180 (11)	−0.0032 (8)	−0.0005 (8)	0.0018 (9)
C21	0.0195 (10)	0.0166 (11)	0.0139 (11)	−0.0027 (8)	0.0035 (8)	−0.0004 (9)
C30	0.0179 (10)	0.0152 (11)	0.0156 (10)	0.0017 (8)	−0.0033 (8)	−0.0015 (9)
C7	0.0189 (10)	0.0219 (12)	0.0126 (11)	−0.0020 (9)	0.0015 (8)	−0.0025 (9)
C27	0.0165 (10)	0.0171 (11)	0.0141 (10)	−0.0011 (8)	−0.0018 (8)	−0.0001 (9)
C24	0.0173 (10)	0.0174 (11)	0.0132 (11)	0.0001 (9)	−0.0003 (8)	0.0006 (9)
C20	0.0206 (10)	0.0163 (11)	0.0174 (11)	0.0048 (9)	0.0004 (8)	0.0002 (9)
C1	0.0186 (10)	0.0170 (11)	0.0132 (11)	−0.0002 (8)	0.0020 (8)	0.0014 (9)
C26	0.0167 (9)	0.0192 (11)	0.0136 (11)	0.0028 (9)	−0.0008 (8)	0.0011 (9)
C6	0.0198 (10)	0.0167 (11)	0.0168 (11)	−0.0040 (9)	0.0003 (8)	0.0007 (9)
C8	0.0167 (10)	0.0169 (11)	0.0199 (12)	−0.0015 (8)	0.0000 (9)	−0.0020 (9)
C3	0.0199 (10)	0.0155 (11)	0.0183 (11)	−0.0046 (8)	0.0020 (8)	−0.0015 (9)
C25	0.0155 (10)	0.0176 (12)	0.0158 (10)	0.0013 (8)	−0.0003 (8)	0.0006 (9)
C23	0.0179 (10)	0.0143 (10)	0.0170 (10)	0.0029 (8)	0.0023 (8)	0.0002 (9)
C29	0.0151 (9)	0.0227 (11)	0.0170 (10)	0.0006 (9)	0.0005 (8)	0.0004 (9)
C10	0.0179 (10)	0.0163 (11)	0.0153 (11)	−0.0007 (8)	0.0010 (8)	−0.0028 (8)
C32	0.0168 (9)	0.0208 (11)	0.0174 (11)	−0.0008 (9)	0.0019 (8)	−0.0010 (9)
C19	0.0149 (10)	0.0209 (12)	0.0165 (11)	0.0024 (8)	−0.0005 (8)	0.0007 (9)
C28	0.0170 (10)	0.0149 (10)	0.0172 (11)	−0.0033 (8)	0.0004 (8)	−0.0001 (9)
C2	0.0157 (10)	0.0226 (12)	0.0149 (11)	−0.0020 (8)	−0.0006 (8)	0.0010 (9)
C9	0.0153 (9)	0.0198 (11)	0.0175 (11)	−0.0021 (8)	0.0007 (8)	−0.0043 (9)
C13	0.0198 (10)	0.0176 (11)	0.0151 (11)	−0.0005 (8)	0.0031 (8)	−0.0015 (8)
C11	0.0166 (10)	0.0190 (11)	0.0178 (11)	0.0034 (9)	−0.0001 (8)	0.0004 (9)
C34	0.0308 (12)	0.0149 (11)	0.0330 (14)	0.0005 (10)	0.0069 (10)	−0.0025 (10)
C12	0.0144 (10)	0.0196 (11)	0.0196 (11)	−0.0009 (8)	−0.0007 (8)	−0.0019 (9)
C14	0.0201 (10)	0.0198 (11)	0.0164 (11)	0.0039 (9)	−0.0015 (8)	0.0012 (9)
C31	0.0189 (10)	0.0172 (11)	0.0186 (11)	−0.0027 (9)	−0.0005 (8)	0.0000 (9)
C15	0.0169 (10)	0.0199 (11)	0.0174 (11)	−0.0010 (8)	−0.0006 (8)	−0.0025 (9)
C17	0.0265 (11)	0.0183 (11)	0.0237 (12)	0.0008 (9)	−0.0007 (10)	0.0043 (9)
C16	0.0235 (11)	0.0207 (12)	0.0352 (14)	−0.0044 (9)	−0.0025 (10)	0.0053 (10)
C33	0.0250 (12)	0.0219 (12)	0.0310 (13)	0.0032 (10)	0.0053 (10)	−0.0032 (10)
N2	0.0196 (9)	0.0182 (10)	0.0333 (11)	−0.0004 (8)	0.0048 (8)	−0.0041 (8)
N1	0.0178 (9)	0.0173 (9)	0.0262 (10)	−0.0007 (8)	−0.0011 (8)	0.0037 (8)

### Geometric parameters (Å, °)

**Table:**

O2—C4	1.363 (3)	C23—H23	0.9300
O2—H2O	0.8200	C29—C28	1.376 (3)
O1—C7	1.239 (3)	C29—H29	0.9300
O4—C21	1.359 (3)	C10—C15	1.398 (3)
O4—H4O	0.8200	C10—C11	1.406 (3)
O3—C24	1.239 (3)	C10—C9	1.456 (3)
C22—C23	1.373 (3)	C32—C31	1.375 (3)
C22—C21	1.395 (3)	C32—H32	0.9300
C22—H22	0.9300	C19—H19	0.9300
C18—C23	1.401 (3)	C28—H28	0.9300
C18—C19	1.402 (3)	C2—H2	0.9300
C18—C24	1.481 (3)	C9—H9	0.9300
C4—C5	1.389 (3)	C13—N1	1.378 (3)
C4—C3	1.395 (3)	C13—C14	1.407 (3)
C5—C6	1.385 (3)	C13—C12	1.415 (3)
C5—H5	0.9300	C11—C12	1.376 (3)
C21—C20	1.400 (3)	C11—H11	0.9300
C30—N2	1.370 (3)	C34—N2	1.448 (3)
C30—C31	1.411 (3)	C34—H34A	0.9600
C30—C29	1.413 (3)	C34—H34B	0.9600
C7—C8	1.469 (3)	C34—H34C	0.9600
C7—C1	1.478 (3)	C12—H12	0.9300
C27—C32	1.399 (3)	C14—C15	1.378 (3)
C27—C28	1.410 (3)	C14—H14	0.9300
C27—C26	1.451 (3)	C31—H31	0.9300
C24—C25	1.464 (3)	C15—H15	0.9300
C20—C19	1.379 (3)	C17—N1	1.454 (3)
C20—H20	0.9300	C17—H17A	0.9600
C1—C2	1.401 (3)	C17—H17B	0.9600
C1—C6	1.402 (3)	C17—H17C	0.9600
C26—C25	1.344 (3)	C16—N1	1.452 (3)
C26—H26	0.9300	C16—H16A	0.9600
C6—H6	0.9300	C16—H16B	0.9600
C8—C9	1.344 (3)	C16—H16C	0.9600
C8—H8	0.9300	C33—N2	1.452 (3)
C3—C2	1.379 (3)	C33—H33A	0.9600
C3—H3	0.9300	C33—H33B	0.9600
C25—H25	0.9300	C33—H33C	0.9600
			
C4—O2—H2O	109.5	C27—C32—H32	119.0
C21—O4—H4O	109.5	C20—C19—C18	121.1 (2)
C23—C22—C21	119.95 (19)	C20—C19—H19	119.4
C23—C22—H22	120.0	C18—C19—H19	119.4
C21—C22—H22	120.0	C29—C28—C27	121.9 (2)
C23—C18—C19	118.2 (2)	C29—C28—H28	119.1
C23—C18—C24	122.4 (2)	C27—C28—H28	119.1
C19—C18—C24	119.43 (19)	C3—C2—C1	121.03 (19)
O2—C4—C5	122.33 (18)	C3—C2—H2	119.5
O2—C4—C3	117.3 (2)	C1—C2—H2	119.5
C5—C4—C3	120.3 (2)	C8—C9—C10	127.8 (2)
C6—C5—C4	119.37 (19)	C8—C9—H9	116.1
C6—C5—H5	120.3	C10—C9—H9	116.1
C4—C5—H5	120.3	N1—C13—C14	120.6 (2)
O4—C21—C22	122.38 (19)	N1—C13—C12	121.82 (19)
O4—C21—C20	117.75 (19)	C14—C13—C12	117.56 (19)
C22—C21—C20	119.9 (2)	C12—C11—C10	121.8 (2)
N2—C30—C31	120.7 (2)	C12—C11—H11	119.1
N2—C30—C29	122.25 (19)	C10—C11—H11	119.1
C31—C30—C29	116.99 (19)	N2—C34—H34A	109.5
O1—C7—C8	121.3 (2)	N2—C34—H34B	109.5
O1—C7—C1	119.2 (2)	H34A—C34—H34B	109.5
C8—C7—C1	119.46 (18)	N2—C34—H34C	109.5
C32—C27—C28	116.7 (2)	H34A—C34—H34C	109.5
C32—C27—C26	120.11 (18)	H34B—C34—H34C	109.5
C28—C27—C26	123.17 (19)	C11—C12—C13	120.82 (19)
O3—C24—C25	121.8 (2)	C11—C12—H12	119.6
O3—C24—C18	119.52 (19)	C13—C12—H12	119.6
C25—C24—C18	118.70 (18)	C15—C14—C13	120.5 (2)
C19—C20—C21	119.6 (2)	C15—C14—H14	119.7
C19—C20—H20	120.2	C13—C14—H14	119.7
C21—C20—H20	120.2	C32—C31—C30	121.2 (2)
C2—C1—C6	118.1 (2)	C32—C31—H31	119.4
C2—C1—C7	118.76 (19)	C30—C31—H31	119.4
C6—C1—C7	123.1 (2)	C14—C15—C10	122.4 (2)
C25—C26—C27	126.26 (19)	C14—C15—H15	118.8
C25—C26—H26	116.9	C10—C15—H15	118.8
C27—C26—H26	116.9	N1—C17—H17A	109.5
C5—C6—C1	121.3 (2)	N1—C17—H17B	109.5
C5—C6—H6	119.3	H17A—C17—H17B	109.5
C1—C6—H6	119.3	N1—C17—H17C	109.5
C9—C8—C7	120.84 (19)	H17A—C17—H17C	109.5
C9—C8—H8	119.6	H17B—C17—H17C	109.5
C7—C8—H8	119.6	N1—C16—H16A	109.5
C2—C3—C4	119.8 (2)	N1—C16—H16B	109.5
C2—C3—H3	120.1	H16A—C16—H16B	109.5
C4—C3—H3	120.1	N1—C16—H16C	109.5
C26—C25—C24	123.03 (19)	H16A—C16—H16C	109.5
C26—C25—H25	118.5	H16B—C16—H16C	109.5
C24—C25—H25	118.5	N2—C33—H33A	109.5
C22—C23—C18	121.2 (2)	N2—C33—H33B	109.5
C22—C23—H23	119.4	H33A—C33—H33B	109.5
C18—C23—H23	119.4	N2—C33—H33C	109.5
C28—C29—C30	121.08 (19)	H33A—C33—H33C	109.5
C28—C29—H29	119.5	H33B—C33—H33C	109.5
C30—C29—H29	119.5	C30—N2—C34	120.61 (18)
C15—C10—C11	116.80 (19)	C30—N2—C33	121.81 (19)
C15—C10—C9	119.14 (19)	C34—N2—C33	117.11 (19)
C11—C10—C9	124.1 (2)	C13—N1—C16	119.52 (19)
C31—C32—C27	122.06 (19)	C13—N1—C17	119.53 (18)
C31—C32—H32	119.0	C16—N1—C17	116.36 (19)
			
O2—C4—C5—C6	179.2 (2)	C21—C20—C19—C18	1.9 (3)
C3—C4—C5—C6	−0.2 (3)	C23—C18—C19—C20	−0.1 (3)
C23—C22—C21—O4	−179.3 (2)	C24—C18—C19—C20	−179.62 (19)
C23—C22—C21—C20	0.4 (3)	C30—C29—C28—C27	−0.1 (3)
C23—C18—C24—O3	−160.0 (2)	C32—C27—C28—C29	0.2 (3)
C19—C18—C24—O3	19.5 (3)	C26—C27—C28—C29	179.8 (2)
C23—C18—C24—C25	21.0 (3)	C4—C3—C2—C1	−1.3 (3)
C19—C18—C24—C25	−159.5 (2)	C6—C1—C2—C3	−0.1 (3)
O4—C21—C20—C19	177.69 (19)	C7—C1—C2—C3	−178.3 (2)
C22—C21—C20—C19	−2.1 (3)	C7—C8—C9—C10	174.2 (2)
O1—C7—C1—C2	−14.6 (3)	C15—C10—C9—C8	−174.3 (2)
C8—C7—C1—C2	162.1 (2)	C11—C10—C9—C8	6.1 (4)
O1—C7—C1—C6	167.3 (2)	C15—C10—C11—C12	−0.6 (3)
C8—C7—C1—C6	−16.0 (3)	C9—C10—C11—C12	179.0 (2)
C32—C27—C26—C25	−165.5 (2)	C10—C11—C12—C13	0.2 (3)
C28—C27—C26—C25	14.9 (4)	N1—C13—C12—C11	180.0 (2)
C4—C5—C6—C1	−1.3 (3)	C14—C13—C12—C11	0.7 (3)
C2—C1—C6—C5	1.4 (3)	N1—C13—C14—C15	179.5 (2)
C7—C1—C6—C5	179.5 (2)	C12—C13—C14—C15	−1.1 (3)
O1—C7—C8—C9	8.5 (3)	C27—C32—C31—C30	1.2 (3)
C1—C7—C8—C9	−168.1 (2)	N2—C30—C31—C32	177.3 (2)
O2—C4—C3—C2	−178.01 (19)	C29—C30—C31—C32	−1.0 (3)
C5—C4—C3—C2	1.4 (3)	C13—C14—C15—C10	0.8 (3)
C27—C26—C25—C24	−175.8 (2)	C11—C10—C15—C14	0.1 (3)
O3—C24—C25—C26	16.2 (4)	C9—C10—C15—C14	−179.5 (2)
C18—C24—C25—C26	−164.8 (2)	C31—C30—N2—C34	−2.0 (3)
C21—C22—C23—C18	1.4 (3)	C29—C30—N2—C34	176.3 (2)
C19—C18—C23—C22	−1.6 (3)	C31—C30—N2—C33	169.9 (2)
C24—C18—C23—C22	177.9 (2)	C29—C30—N2—C33	−11.8 (3)
N2—C30—C29—C28	−177.9 (2)	C14—C13—N1—C16	−174.7 (2)
C31—C30—C29—C28	0.5 (3)	C12—C13—N1—C16	6.0 (3)
C28—C27—C32—C31	−0.8 (3)	C14—C13—N1—C17	−19.6 (3)
C26—C27—C32—C31	179.6 (2)	C12—C13—N1—C17	161.1 (2)

### Hydrogen-bond geometry (Å, °)

**Table:**

*D*—H···*A*	*D*—H	H···*A*	*D*···*A*	*D*—H···*A*
O4—H4O···O1^i^	0.82	1.85	2.670 (2)	173
O2—H2O···O3^ii^	0.82	1.85	2.659 (2)	169

Symmetry codes: (i) *x*−1, *y*, *z*; (ii) *x*−1, *y*, *z*−1.
